# Splicing Analysis of Exonic *OCRL* Mutations Causing Lowe Syndrome or Dent-2 Disease

**DOI:** 10.3390/genes9010015

**Published:** 2018-01-04

**Authors:** Lorena Suarez-Artiles, Ana Perdomo-Ramirez, Elena Ramos-Trujillo, Felix Claverie-Martin

**Affiliations:** Unidad de Investigación, Hospital Nuestra Señora de Candelaria, 38010 Santa Cruz de Tenerife, Spain; lsartiles88@gmail.com (L.S.-A.); atter_rad@hotmail.com (A.P.-R.); eramostrujillo@gmail.com (E.R.-T.)

**Keywords:** *OCRL* gene, exonic mutation, splicing mutation, missense mutation, splice defects, exon skipping, minigene assay, bioinformatics tools, Lowe syndrome, Dent-2 disease

## Abstract

Mutations in the *OCRL* gene are associated with both Lowe syndrome and Dent-2 disease. Patients with Lowe syndrome present congenital cataracts, mental disabilities and a renal proximal tubulopathy, whereas patients with Dent-2 disease exhibit similar proximal tubule dysfunction but only mild, or no additional clinical defects. It is not yet understood why some *OCRL* mutations cause the phenotype of Lowe syndrome, while others develop the milder phenotype of Dent-2 disease. Our goal was to gain new insights into the consequences of *OCRL* exonic mutations on pre-mRNA splicing. Using predictive bioinformatics tools, we selected thirteen missense mutations and one synonymous mutation based on their potential effects on splicing regulatory elements or splice sites. These mutations were analyzed in a minigene splicing assay. Results of the RNA analysis showed that three presumed missense mutations caused alterations in pre-mRNA splicing. Mutation c.741G>T; p.(Trp247Cys) generated splicing silencer sequences and disrupted splicing enhancer motifs that resulted in skipping of exon 9, while mutations c.2581G>A; p.(Ala861Thr) and c.2581G>C; p.(Ala861Pro) abolished a 5′ splice site leading to skipping of exon 23. Mutation c.741G>T represents the first *OCRL* exonic variant outside the conserved splice site dinucleotides that results in alteration of pre-mRNA splicing. Our results highlight the importance of evaluating the effects of *OCRL* exonic mutations at the mRNA level.

## 1. Introduction

Altered pre-mRNA splicing is currently recognized as the underlying cause of many hereditary diseases [1,2,3,4]. Present knowledge reveals that, in addition to their protein coding potential, exonic sequences are also involved in the regulation of pre-mRNA splicing. Exons may contain splicing regulatory elements such as exonic splicing enhancers (ESEs) and exonic splicing silencers (ESSs) that promote or inhibit the recognition of the neighboring splice sites, respectively [1]. Also, the last three positions and the first two in the exons are an integral part of the 5′ and 3′ splice sites consensus sequences, respectively. Therefore, exonic mutations may affect splicing by altering regulatory elements, by abolishing or reducing the strength of the splice sites, or by creating new splice sites [1,5].

Lowe syndrome (OMIM #309000) and Dent disease type-2 (Dent-2; OMIM #300555) are two rare X-linked renal tubulopathies progressing to chronic kidney failure [6,7,8]. Lowe syndrome, a severe disorder, is also characterized by congenital cataracts, mental disabilities, and hypotonia. Dent-2 disease shows no, or only mild, additional clinical defects suggesting that Dent-2 represents a mild form of Lowe syndrome [9,10]. The dysfunction of the renal proximal tubule results in urinary loss of low-molecular weight proteins, phosphate and bicarbonate [11]. Similar renal tubular defects are also characteristics of Dent-1, another X-linked disease that is caused by mutations in the gene *CLCN5* that encodes for the endosomal proton-chloride exchanger ClC-5 [6]. Lowe syndrome and Dent-2 disease are caused by mutations in the *OCRL* gene located on chromosome Xq26.1 [12,13]. This gene encodes OCRL1, an inositol polyphosphate 5-phosphatase that preferentially hydrolyses phosphatidylinositol 4,5-bisphosphate, a key regulator in intracellular trafficking [12,14]. The full-length *OCRL* mRNA transcript contains 24 exons, and produces two splice isoforms, called A and B [15]. Isoform B lacks exon 19 (also referred to as 18a) encoding 8 amino acids that is present in isoform A (901 amino acids). Isoform A is ubiquitously expressed while the B isoform is expressed in all tissues except the brain. Isoform A binds clathrin with higher affinity than isoform B and is involved in endocytosis of low-molecular weight proteins and intracellular membrane traffic in the proximal tubule [16]. The OCRL1 protein contains several domains, including an N-terminal pleckstrin homology (PH) domain, a central 5-phosphatase catalytic domain, an ASH (ASPM, SPD-2, Hydin) domain, and a C-terminal noncatalytic Rho-GTPase activating protein (GAP) domain [8,17]. In addition, OCRL1 contains several protein binding motifs that facilitate its interaction with different proteins, which mostly assist its targeting to different cell compartments such as the plasma membrane, trans-Golgi network, early endosomes, clathrin-coated vesicles, primary cilium and lysosomes [8,17].

According to the Human Gene Mutation Database (HGMD) [18], approximately 250 *OCRL* disease-causing mutations have been described, including missense and nonsense mutations (49%), splicing mutations (12%), small deletions (20%), and small insertions (9%), and gross deletions and insertions. Missense and nonsense mutations occur throughout the *OCRL* gene, but mainly in exons 9 to 24 that contain the 5-phosphatase domain, the ASH domain, and the RhoGAP domain [8,19,20]. It is not yet understood why some *OCRL* mutations cause the phenotype of Lowe syndrome, while others develop the milder phenotype of Dent-2 disease. Also, we know that in Dent-2 disease nonsense mutations cluster in exons 4 to 8. Therefore, it would be interesting to investigate these *OCRL* missense and nonsense mutations for their potential effect on pre-mRNA splicing.

The aim of our study was to gain insights into the consequences of previously described *OCRL* missense and synonymous mutations on pre-mRNA splicing. A total of 45 mutations located mainly in exons 9, 11, 12, 15, 21, 22 and 23 were analyzed with bioinformatics tools. Thirteen missense mutations and one synonymous mutation affecting splicing regulatory elements or splice sites were selected and their mRNAs were studied using a minigene system.

## 2. Materials and Methods 

The nomenclature for the description of mutations followed the guidelines of the Human Genome Variation Society [21]. Nucleotide numbering was based on the *OCRL* cDNA sequence (GenBank accession number NM_000276.3), with c.1 denoting the first position of the translation start codon.

### 2.1. Bioinformatics Predictions

All missense and synonymous *OCRL* mutations were selected from the HGMD [18], except c.1070G>T, c.1567G>T, c.2418G>T and c.2581G>C [22], and c.1221G>A, p.(Pro407Pro), that was identified in one of our patients [23]. In silico analysis were performed with different web-based bioinformatics tools in order to select mutations with potential effect in pre-mRNA processing. Splice site prediction by neural network (NNSplice) was used to analyze exon definition and to predict the putative effect of mutations on the consensus splice site regions [24]. To identify the presence of potential splicing regulatory sequences such as ESEs and ESSs, and determine the putative effect of mutations on splicing regulatory motifs, we used ESEfinder 3.0 [25], RESCUE-ESE [26], and FAS-ESS [27]. Additionally, we used two new tools, MutPred Splice v1.3.2 [28] and splicing-based analysis of variants (SPANR) [29] to identify exonic mutations possibly affecting pre-mRNA splicing and to determine whether mutations can cause splicing misregulation leading to disease. For the experimental analysis of our study, we selected *OCRL* mutations based on the following criteria: (a) location of mutations in poorly defined exons (exons that have 5′ or 3′ splice sites with NNSplice score below 0.85); (b) predicted activation of a cryptic splice site within the exon; (c) predicted effect of the mutation on the natural 5′ or 3′ splice sites; (d) predicted effect the mutation on exonic splicing regulatory elements (loss of ESEs or/and gain of ESSs).

### 2.2. Amplification of OCRL Genomic Fragments

*OCRL* genomic segments encompassing exons 9 and 10 and intron 9 (179 bp upstream of exon 9 and 62 bp downstream of exon 10), exons 11 and 12 and intron 11 (155 bp upstream of exon 11 and 138 bp downstream of exon 12), exon 15 (118 bp upstream of the exon and 330 bp downstream of the exon), exons 21 to 23 and introns 21 and 22 (115 bp upstream of exon 21 and 117 bp downstream of exon 23) and the corresponding flanking intronic sequences were amplified by PCR from genomic DNA extracted from peripheral blood of a healthy control. PCR reactions were carried out using AccuTaq LA polymerase (Sigma-Aldrich, Saint Louis, MO, USA), and forward and reverse primers. These primers contained at their 5′ ends sequences for restriction enzymes *XhoI* and *XbaI* or *BamHI* (Thermo Fisher Scientific, Waltham, MA, USA) for directional cloning. Polymerase chain reaction (PCR) products were purified using GenElute™ PCR Clean-Up kit (Sigma-Aldrich). Primers were designed using the web-based sources Primer3 [30] and Primer BLAST [31], and the software Gene Runner [32]. Primer sequences are shown in Table S1. Extraction of DNA from the healthy control was carried out with the written consent of the subject and was approved by the Clinical Research Ethics Committee of Hospital Nuestra Señora de la Candelaria, Santa Cruz de Tenerife, Spain.

### 2.3. Generation of Minigene Constructs

Amplified fragments were cloned into the expression vector Exontrap (pET01, MoBiTech, Göttingen, Germany). Fragments and vector were separately digested using the appropriate combination of restriction enzymes. Ligation reactions were performed using T4 DNA ligase (Kapa Biosystems, Wilmington, MA, USA) according to the manufacturer’s instructions. XL1 Blue competent cells were transformed with 5 μL of reaction product by heat-shock and grown in Luria Broth (LB) agar plates supplemented with ampicillin. Colonies carrying recombinant plasmids were grown overnight in LB with ampicillin at 37 °C. Plasmid DNA extraction was carried out with GenElute™ Plasmid Miniprep Kit (Sigma-Aldrich), and recombinant plasmids were confirmed by double digestion with the same restriction enzymes used for cloning. Minigenes were analyzed by sequencing the entire *OCRL* insert with BigDye^®^ Terminator v3.1 Cycle Sequencing Kit (Thermo Fischer Scientificusing primers ETprim04 and ETprim05 (MoBiTech) that anneal to vector pET01 sequences.

### 2.4. Site-Directed Mutagenesis

Mutations of interest were introduced in the minigenes using the QuickChange^®^ Lightning Site-Directed Mutagenesis Kit (Agilent Technologies, Santa Clara, CA, USA) following the manufacturer’s recommendations. Reaction products were transformed into XL10-Gold ultracompetent cells. Primers for mutagenesis were designed using the bioinformatics tool QuickChange^®^ Primer Design Program [33] according to the guidelines described in the QuickChange^®^ commercial kit (Table S1). To confirm the presence of the desired mutation, all constructs were analyzed by directed sequencing using the primers designed for the amplification of each fragment.

### 2.5. Cell Culture, Transient Transfection and RT-PCR Assay

COS7 cells were cultured in Dulbecco’s Modified Eagle Medium (DMEM, Sigma-Aldrich) with low glucose (1 g/L), supplemented with 10% fetal bovine serum and 1% penicillin/streptomycin and incubated at 37 °C and 5% CO_2_ in a humidified incubator. Transfections with wild-type and mutant minigenes were carried out using JetPRIME^®^ reagent (Polyplus Transfection, Illkirch, France) following the recommendations of the manufacturer. Transfected cells were incubated for 48 h, and total RNA was extracted using the High Pure RNA Isolation kit (Roche, Indianapolis, IN, USA). RNA was quantified using the Nanodrop Lite (Thermo Scientific). cDNA synthesis was performed with iScript cDNA synthesis kit (Bio-Rad, Hercules, CA, USA) using random primers. cDNAs were amplified by PCR using primers ETprim02 and ETprim03 (MoBiTech) complementary to sequences of the 5′ and 3′ pET01 exons. Reverse transcription (RT)-PCR products were analyzed by agarose gel electrophoresis together with molecular weight marker DirectLoad PCRbp Low Ladder (Sigma-Aldrich). Products were recovered from the gel using GenElute™ Agarose Spin Columns (Sigma-Aldrich) and sequenced as previously described. DNA sequences were compared to the reference *OCRL* sequence of (GenBank entry number NM_000276.3) using the bioinformatics online program Basic Local Alignment Search Tool (BLAST) [34]. The exact size of each product was determined from the DNA sequence.

## 3. Results

In many instances in which pre-mRNA splicing is affected by an exonic mutation, the exon that is involved appears to be weakly defined [1]. Also, it is known that ESE elements are commonly present in exons with weak splice sites [35]. Therefore, we sought to select *OCRL* mutations located in exons that have a weak 5′ or 3′ splice site (those with a NNSplice score below 0.85). According to the analysis performed with the bioinformatics tool NNSplice, *OCRL* exons 9, 11, 12, 15, 21, 22 and 23 were considered as weak or poorly defined exons (Table S2). A total of 45 mutations affecting nucleotides located on the cited exons were analyzed using the bioinformatics tools FAS-ESS, ESE Finder, Rescue ESE and MutPred Splice (Table S2). 

**Figure 1:**
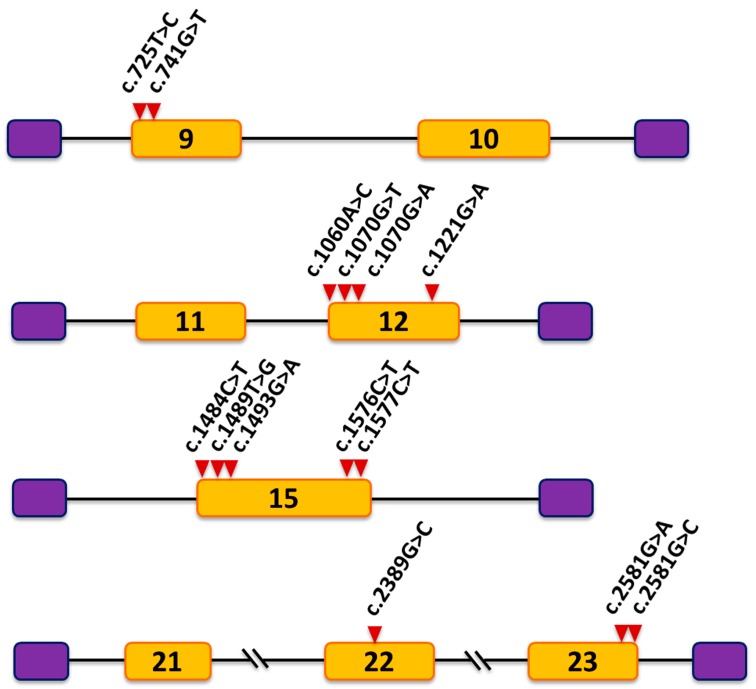
Schematic representation of the four minigenes constructed with expression vector pET01 and *OCRL* wild-type sequences (yellow boxes) containing exons 9-10 (pET-Ex9-10), 11-12 (pET-Ex11-12), 15 (pET-Ex15) and 21-22-23 (pET-Ex21-23), respectively. Purple boxes depict pET01 exons and horizontal lines in between indicate *OCRL* intron sequences. The different mutations introduced by site-directed mutagenesis in each minigene are shown.

From the results of these in silico analyses we selected thirteen mutations (12 missense and 1 synonymous) for minigene analysis, including those that involved the generation of an ESS site or the alteration of an ESE site, the activation of a cryptic splice site, or the loss of a natural 5′ splice site (Table 1). For the minigene analysis, we also included variant c.2389G>C located in the middle of exon 22. Although this variant did not meet the criteria mentioned above, we included it because it could be informative as a negative control. It is worth mentioning that exon 22 has a very weak natural 3′ splice site (NNsplice score of 0.05, Table S2). On the other hand, variant c.1598T>G in exon 15 was selected because it was predicted as a splicing affecting variant (SAV) by MutPred Splice (Table S2); however, regrettably we could not create the corresponding nucleotide change in the minigene. Four different control minigenes were generated comprising *OCRL* wild-type sequences of exons 9-10 (pET-Ex9-10), 11-12 (pET-Ex11-12), 15 (pET-Ex15) and 21-22-23 (pET-Ex21-23) respectively (Figure 1). To construct these minigenes, PCR products were cloned into the pET01 vector as described previously [36]. All the mutations studied were introduced into the minigenes by site-directed mutagenesis using their corresponding control constructs as a template.

### 3.1. Mutation c.741G>T Prevents Incorporation of Exon 9 into the Mature mRNA

**Figure 2:**
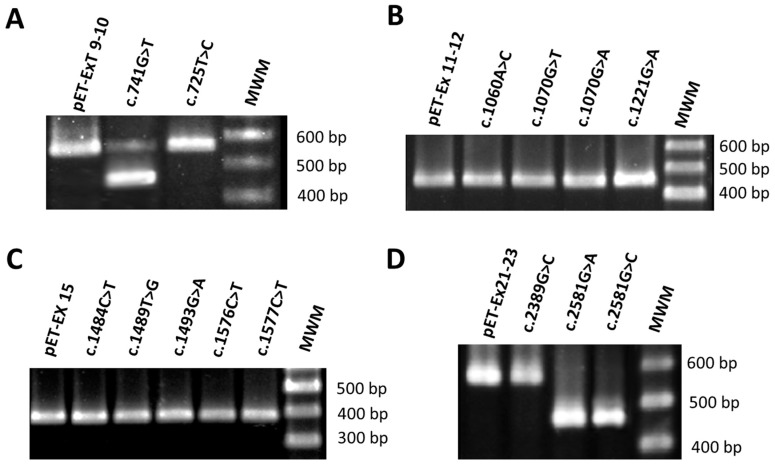
RT-PCR analysis of the spliced transcripts expressed from the *OCRL* minigenes containing wild-type and mutant exons. (**A**) Of the two mutations introduced in exon 9, only c.741G>T; p.(Trp247Cys) generated an altered mRNA product; (**B**,**C**) None of the mutations introduced in exons 12 and 15 showed alterations in splicing; (**D**) Mutations c.2581G>A; p.(Ala861Thr) and c.2581G>C; p.(Ala861Pro) in exon 23 resulted in altered mRNAs. MWM: Molecular weight marker.

**Figure 3:**
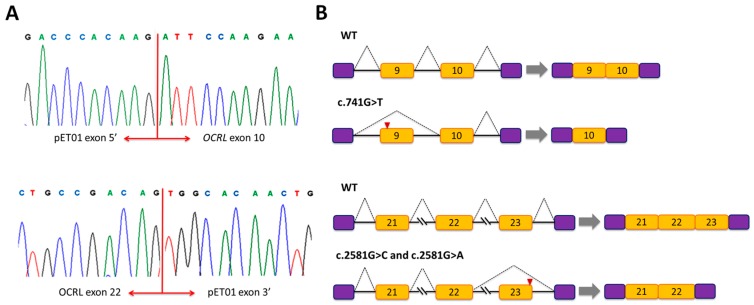
(**A**) DNA sequencing results of the altered RT-PCR products obtained with mutations c.741G>T; p.(Trp247Cys) (top panel) and with mutations c.2581G>A; p.(Ala861Thr) and c.2581G>C; p.(Ala861Pro) (bottom panel) showing the joining of exon 5 of the vector′ with the 5′ end of *OCRL* exon 10, and the joining of the 3′ end of *OCRL* exon 22 with exon 3′ of the vector (**B**) Schematic representation of pre-mRNA splicing in wild-type (WT) and mutants minigenes. Exon 9 and exon 23 are missing in the mRNA from c.741G>T and c.2581G>A /c.2581G>C mutants, respectively.

Mutation c.741G>T; p.(Trp247Cys) is located at position +19 from the 5′ end of exon 9 within a sequence containing two potential overlapping ESE sites, UUG**G**AA and UG**G**AAU (according to Rescue-ESE, affected nucleotide in bold face). Bioinformatics analysis indicated that the mutation eliminates these ESEs. To investigate the effect of mutation c.741G>T, we created a minigene composed of exons 9 and 10, intron 9, and flanking intronic sequences (Figure 1). The wild-type and mutant minigenes were transfected separately into COS7 cells, and RT-PCR analysis was performed. The RT-PCR products obtained from the RNA were examined by agarose gel electrophoresis. The results revealed a different electrophoresis band pattern in the wild-type and mutant minigenes. The wild-type minigene generated the expected splicing product with a size of 467 bp whereas the mutant minigene produced two splicing products: a smaller band of 365 bp and a faint larger band corresponding to the wild-type product (Figure 2). Sequencing analysis showed that the smaller product did not contain exon 9 of *OCRL* but the 5′ exon of pET01 followed by *OCRL* exon 10 and the 3′ exon of pET01 (Figure 3). Examination of the complementary DNA (cDNA) sequence indicated that absence of exon 9 did not result in alteration of the open reading frame. The OCRL1 protein encoded by this altered mRNA would be missing 34 amino acids of the central 5-phosphatase domain (residues 241 to 275). In order to better understand the cause of this splicing defect, we decided to examine further the surrounding sequence that includes mutation c.741G>T. We utilized Human Splicing Finder v3.0 (HSF) [43], a tool that integrates different existing matrices to identify exonic and intronic motifs, including ESS sites. The results of this analysis showed that c.741G>T also generates four overlapping silencer motif sequences, CUUG**U**AAU, UG**U**AAUGU, G**U**AAUG and **U**AAUGU, which correspond to the putative binding site for the splicing repressor heterogeneous nuclear ribonucleoprotein A1 (hnRNPA1), a protein that usually binds to ESSs and inhibits exon recognition. We postulate that an ESS element is created by mutation c.741G>T that would recruit the hnRNPA1 protein and trigger the exon 9 skipping observed. It is worth noting that mutation c.725T>C located also in exon 9 at position +3 relative to the 3′ splice site did not have any effect on pre-mRNA splicing (Figure 1 and Figure 2). 

A properly spliced transcript was observed in the minigene containing mutation c.725T>C; p.(Phe242Ser) located in exon 9 (Figure 2). This mutation affects the third nucleotide of the exon, further upstream from the ESS motif generated by mutation c.741G>T, and according to MutPred Splice was a splice-affecting variant. However, analysis of c.725T>C with HSF showed no significant splicing motif alteration. Therefore, this mutation has probably no impact on pre-mRNA splicing. 

### 3.2. Mutations c.2581G>A and c.2581G>C Result in Skipping of Exon 23

Putative *OCRL* missense mutations c.2581G>A; p.(Ala861Thr) and c.2581G>C; p.(Ala861Pro) result from substitutions at the last nucleotide of exon 23 upstream of the 5′ splice site. Bioinformatics analysis with NNSplice revealed that both mutations drastically decreased the score of the wild-type 5′ splice site (AUGAUC**G**guaa, the nucleotide affected by the mutations appears in bold face, and the conserved GU dinucleotide of the 5′ splice site in intron 23 is underlined) from 0.99 to 0.36 (AUGAUC**A**guaa) and 0.75 (AUGAUC**C**guaa), respectively (Table S2). Analysis of both mutations with MutPred Splice predicted the loss of the 5′ splice site (Table 1). Taken together, these results suggested that exon 23 splicing is altered by these mutations. To determine the experimental effect of mutations c.2581G>A and c.2581G>C we used a minigene containing exons 21, 22 and 23, introns 21 and 22 and flanking intronic sequences (Figure 1). The results of the RT-PCR analysis showed a unique product of 463 bp in both mutant minigenes and a larger band of 575 bp produced by the wild-type minigene (Figure 2). Direct sequencing of these products confirmed that the smaller fragments correspond to skipping of exon 23 in the mRNA, and that the wild-type construct product corresponds to correctly spliced exons (Figure 3). The absence of exon 23 in the spliced mRNA would lead to an aberrant joining of exons 22 and 24 that at the protein level causes the loss of 37 amino acids (residues 824 to 861), a frameshift and the introduction of a premature stop codon nine positions downstream in exon 24. This would eliminate part of the Rho-GAP domain located at the carboxy terminus of the OCRL1 protein. It is also worth noting that, in general, premature stop codons within the last exon of a gene do not activate nonsense-mediated mRNA decay and yield stable mRNAs that direct the synthesis of truncated polypeptides [44].

### 3.3. Mutations in Exons 12, 15 and 22 Did Not Alter Pre-mRNA Splicing

Analysis of the minigenes containing mutations c.1060A>C; p.(Asn354His), c.1070G>T; p.(Gly357Val), c.1070G>A; p.(Gly357Glu), c.1221G>A; p.(Pro407Pro) (exon 12), c.1484C>T; p.(Pro495Leu), c.1489T>G; p.(Trp497Gly), c.1493G>A; p.(Cys498Tyr), c.1576C>T; p.(Pro526Ser), c.1577C>T; p.(Pro526Leu) (exon 15) and mutation c.2389G>C; p.(Ala797Pro) (exon 22) resulted in RT-PCR products that matched in size to those generated by the respective wild-type constructs (Figure 2). This was also confirmed by DNA sequencing. Therefore, these variants did not affect pre-mRNA splicing. As these results were in disagreement with the bioinformatics predictions, we used a recently published computational approach, SPANR [29], to analyze the mutations. This tool extracts more than a thousand features from each exon, its flanking introns, and its adjacent exons, and uses a computational model to predict with great precision if a mutation alters the percentage of transcripts with the exon spliced in. The results showed that none of these ten mutations would lead to a decrease of mRNAs containing the respective exons in contrast with the wild-type sequences (Table 1). Whereas as expected, the three *OCRL* mutations that altered pre-mRNA splicing in the minigene system, c.741G>T, c.2581G>A and c.2581G>C, were predicted to result in a reduction of transcripts containing exons 9 and 23, respectively. Interestingly, the SPANR approach predicted that mutation c.725T>C increases RNA transcripts containing exon 9.

## 4. Discussion

Defects in pre-mRNA splicing play a main role in the development of many genetic diseases [2,4]. It is still not very well known how large is the fraction of disease-causing mutations that lead to splicing alterations. Estimates ranging from 15% to 50% are often cited in the literature [45,46,47]. These mutations affect splicing not only by disrupting splice sites, but also by altering splicing regulatory sequences or by creating new splice sites [1,2]. The important fraction of splicing mutations reflects the necessity to characterize mutations at the mRNA level since exonic mutations outside the conserved splice site dinucleotides could certainly be misclassified as missense or synonymous mutations if only the DNA is examined. 

Several bioinformatics tools can be used to predict potential consequences of mutations on pre-mRNA splicing. Those used to predict the effect of splice site mutations, such as NNSplice and HSF [24,43], have a good performance since the consensus splice site regions are well established. Conversely, evaluating precisely how mutations influence the loss or gain of splicing regulatory elements (ESEs or ESSs) and its effects on pre-mRNA splicing is more complicated, and the algorithms available, like RESCUE-ESE, ESEfinder and MutPred Splice [25,26,28], still present considerable degrees of inaccuracy [48,49,50]. Therefore, their findings need to be confirmed using experimental methods. The ideal experimental approach to evaluate the effect of mutations on pre-mRNA splicing is to analyze the RNA from the patient. However, it is not always possible to obtain these kinds of samples. 

A functional splicing assay based on a minigene construct represents an interesting alternative when patients’ samples are not available [51]. In this approach a genomic fragment, containing the exon with the mutation of interest and adjacent intronic sequences, is amplified and cloned into a minigene vector. After transient transfection into cultured cells, the splicing patterns of the mRNAs produced by wild-type and mutant constructs are compared by RT-PCR analysis and DNA sequencing. The effectiveness of minigene systems has been confirmed by different studies that showed a high level of concordance between results obtained with these assays and results from patient’s RNA [52,53,54,55,56,57]. In previous studies, we have used this system to assess the consequences on pre-mRNA splicing of presumed missense and synonymous *PKD1* and *PKD2* mutations, and found that six of them were indeed splicing mutations [36,58]. 

Single-base substitutions in the *OCRL* gene, many of which are predicted to lead to missense mutations, are often found in patients with Lowe syndrome or Dent-2 disease [19,22]. Here, we assumed that the primary pathogenic effect for some of these *OCRL* mutations is at the level of pre-mRNA splicing. Using bioinformatics tools, we selected 12 missense and 1 synonymous variants located in exons 9, 12, 15, 22 and 23 and tested them with a minigene assay. These mutations were predicted to modify splicing regulatory sequences (disrupt ESEs or create ESSs), generate new splice sites or induce a significant reduction of splice site strength. We constructed minigene plasmids ligated to either wild-type or mutant *OCRL* genomic sequences spanning exons 9 to 10, 11 to 12, 15 and 21 to 23. The constructs were transfected into COS7 cells, and the mRNA was analyzed by RT-PCR and DNA sequencing. This approach allowed us to categorize three exonic *OCRL* mutations associated with Lowe syndrome, c.741G>T; p.(Trp247Cys), c.2581G>A; p.(Ala861Thr) and c.2581G>C; p.(Ala861Pro) as splicing mutations. Our results showed that mutation c.741G>T prevents exon 9 inclusion in the mRNA probably by affecting a functional ESE site and/or creating a functional ESS site that binds hnRNPA1. The bioinformatics analysis predicted loss of two ESE and gain of four ESS motifs. We hypothesized that the ESE sequences affected by this mutation are necessary for correct splicing since the first nucleotide of exon 9 is A (instead of the conserved G) and the −3 position of intron 8 is a U (instead of the more common C), which weakens the 3′ splice site of intron 8. The mutation would allow the binding of the repressor, causing exon skipping. Unfortunately, the patient’s RNA was not available for analysis. Therefore, it remains to be determined if *OCRL* mutation c.741G>T has the same effect in the context of its natural gene using patient-derived RNA. Mutations in other genes associated with different diseases have been shown to have similar effects [58,59,60,61,62].

Transcript analysis by quantitative PCR of three *OCRL* mutations (c.824G>C, c.1466G>A, c.2581G>A), involving the last nucleotide of exons 9, 14 and 23, has shown that they affect pre-mRNA splicing [19]. Mutations c.824G>C and c.1466G>A were initially reported as missense mutations p.(Gly275Ala) and p.(Ser489Asn), respectively, while c.2581G>A was predicted both as splice mutation and missense p.(Ala861Thr). In fact, these are the only *OCRL* exonic mutations of this type described thus far. Our NNSplice analysis of mutations c.2581G>A and c.2581G>C showed a reduction in the score of the 5′ splice site (Table S2). The minigene analysis of these two mutations revealed that they certainly caused skipping of the entire exon 23. Similar cases have been described in the literature. For instance, the substitution c.2103G>C, in the last position of exon 18 of *MLH1*, does not give rise to a missense mutation (p.(Gln701His)) as hypothetically predicted, but leads to a total loss of exon 18 in the mRNA [45]. Moreover, mutations in the first position of an exon, such as *BRCA2* mutation c.517G>T located in exon 7, also causes exon skipping [63]. The minigene splicing assay for presume missense mutation c.741G>T, p.(Trp247Cys), showed an aberrant transcript that lacks exon 9. We hypothesize that this is probably due to the disruption of a functional ESE site and/or the generation of a functional ESS site, which binds hnRNPA1. Although the main RT-PCR product for this mutant was the transcript lacking exon 9, the effect on pre-mRNA splicing was only partial since it still produced some transcripts with the size of the wild-type product. Therefore, variant c.741G>T probably has a double damaging effect; while the major part of the *OCRL* transcript is deleterious due to exon 9 skipping, which would cause loss of part of the 5′-phosphatase catalytic domain in the mutated OCRL1 protein, the remaining mRNA is damaging due to the resulting amino acid change of Trp to Cys in position 247 of the polypeptide, which could lead to a non-functional OCRL1 protein. The splicing effect of mutation c.741G>T needs to be confirmed analysing the mRNA from the patient, which we did not have. As far as we know, this mutation has been described in only one patient with Lowe syndrome [37], and there is no additional evidence for its pathogenicity. Consequently, in the absence of further information, c.741G>T should be considered as a variant of unknown clinical significance [64].

Eleven of the fourteen *OCRL* mutations tested with the minigene system did not produce any aberrant transcripts, even though the bioinformatics tools we used (ESEfinder, RESCUE-ESE, FASS-ESS and MutPred Splice) predicted gain of ESSs, loss of ESEs and/or generation of cryptic 5′ splice sites (Table 1). This indicated poor concordance between the in silico predictions and the experimental results. Conversely, the SPANR tool applied retrospectively to analyze the selected variants was more accurate than the other tools showing 100% concordance with the experimental results. Notably, the retrospective SPANR analysis of the remaining 31 variants yielded 7 that were predicted to result in reduction of transcripts containing the corresponding exons. From our results, the use of the SPANR approach seems appropriate for the selection of *OCRL* exonic mutations with potential effect in pre-mRNA splicing. The other in silico tools rendered many false positives and seemed unsuitable for the pre-selection of variants. Recently, Soukarieh et al. used experimental data from both minigene splicing assays and analysis of patient’s RNA to compare the performance of SPANR and two other in silico splice-affecting variant predictors [65]. The results of this study showed that the other two tools outperformed SPANR identifying exonic splicing mutations in specific exons of *BRCA1*, *BRCA2*, *MLH1*, *CFTR* and *NF1*. However, the authors could not exclude that the SPANR method may be suitable for the analysis of other exons or genes.

Knowledge of the consequences of exonic splicing mutations may have potential therapeutic implications for patients with Lowe syndrome. Exon-skipping approaches to correct mutations that disrupt normal pre-mRNA splicing have been effectively assessed in several rare diseases [66]. Recently, Rendu et al. designed a successful exon-skipping strategy able to restore significant levels of *OCRL* mRNA and protein in a Lowe syndrome patient with an intronic mutation that induces incorporation of intronic sequences in the mRNA and leads to loss of OCRL1 protein [67]. 

In summary, we have carried out an extensive analysis of exonic *OCRL* mutations using bioinformatics tools and minigenes. The results indicate that mutations c.741G>T, c.2581G>A and c.2581G>C cause significant pre-mRNA splicing alterations which should be taken into account with regard to their pathogenicity. Presume missense mutation c.741G>T; p.(Trp247Cys) affects ESE elements, and at the same time generates ESS motifs that are potential binding sites for the splicing repressor hnRNPA1. This mutation represents the first *OCRL* exonic variant outside the consensus splice site regions that leads to alteration of pre-mRNA splicing. We propose that mutation c.741G>T should be classified as a splicing mutation. These results highlight the importance of evaluating the effects of missense and synonymous mutations at the mRNA level in Lowe syndrome. 

## Figures and Tables

**Table 1:** Mutations analyzed with the minigene system.

Mutation	Reference	Disease	Exon	Position ^1^	FAS-ESS	ESE Finder	Rescue ESE	MutPred Splice ^3^		SPANR	Splice Effect Observed
					Gained ESS ^2^	Disrupted ESE ^2^		Result Confident Hypothesis		PSI	
c.725T>C p.(Phe242Ser)	[19]	Lowe S.	9	+3	0	0	0	SAV	---	Increased	None
c.741G>T p.(Trp247Cys)	[37]	Lowe S.	9	+19	0	0	2	SAV	ESE loss, ESS gain	Decreased	Exon 9 skipping
c.1060A>C p.(Asn354His)	[19]	Dent-2	12	+4	0	1	2	SAV	---	---	None
c.1070G>T p.(Gly357Val)	[22]	Lowe S.	12	+14	0	0	0	SAV	Cryptic 5′ SS	---	None
c.1070G>A p.(Gly357Glu)	[38]	Lowe S.	12	+14	1	0	0	SAV	ESS loss, Cryptic 5′ SS	---	None
c.1221G>A p.(Pro407Pro)	This study	Dent-2	12	−24	0	1	0	SNV	---	---	None
c.1484C>T p.(Pro495Leu)	[39]	Lowe S.	15	+18	2	3	0	SAV	ESE loss, ESS gain	---	None
c.1489T>G p.(Trp497Gly)	[40]	Lowe S.	15	+23	2	0	0	SAV	ESE loss, ESS gain	---	None
c.1493G>A p.(Cys498Tyr)	[38]	Lowe S.	15	+27	0	0	0	SAV	Cryptic 5′ SS	---	None
c.1576C>T p.(Pro526Ser)	[41]	Dent-2	15	−27	0	0	0	SAV	ESE loss	---	None
c.1577C>T p.(Pro526Leu)	[42]	Lowe S.	15	−26	1	0	0	SAV	ESE loss, ESS gain	---	None
c.2389G>C p.(Ala797Pro)	[38]	Lowe S.	22	+48	0	0	0	SNV	---	---	None
c.2581G>A p.(Ala861Thr)	[11]	Lowe S.	23	−1	0	0	0	SAV	Loss of natural 5′ SS	Decreased	Exon 23 skipping
c.2581G>C p.(Ala861Pro)	[22]	Lowe S.	23	−1	0	0	0	SAV	Loss of natural 5′ SS	Decreased	Exon 23 skipping

SAV, Splicing Affecting Variant; SNV, Splicing Neutral Variant; ESE, Exonic Splicing Enhancer; ESS, Exonic Splicing Silencer; FAS-ESS, fluorescence-activated screen for ESS; SS, Splice Site; SPANR, Splicing-based Analysis of Variants; PSI, Percentage of transcripts with the exon spliced in; (---), No predicted effect. ^1^ Position of variant relative to the nearest splice site. ^2^ Values 0, 1, 2 and 3 indicate number of splicing regulatory elements gained or disrupted. ^3^ Score ≥ 0.6 corresponds to SAV; additional supporting evidence is provided by a “confidence hypothesis” which is not available for all SAVs.

## Supplementary Materials

The following are available online at www.mdpi.com/2073-4425/9/1/15/s1. Table S1. Primers used in construction of minigenes and site-directed mutagenesis, Table S2. Mutations analyzed with bioinformatics tools.
