# Supplementary material for: Splicing Analysis of Exonic OCRL Mutations Causing Lowe Syndrome or Dent-2 Disease
Source: Genes (Basel). 2018 Jan 4;9(1):15. doi: 10.3390/genes9010015 (PMC5793168; doi:10.3390/genes9010015)
Supplement: Supplementary file 1 [file genes-09-00015-s001.pdf]

Table S1. Primers used in construction of minigenes and site-directed mutagenesis

**Exons**	**Primer (5′-3′)**	
	PCR amplification	*PCR product size* **(bp)**
**9-10 F**	ATTA*CTCGAG*AAGCCCACTGCCTTTGTGTTC	
**9-10 R**	TATA*GGATCC*GAATAACTCCCGGTGAGCCC	985
**11-12F**	TAAA*CTCGAG*AGTTTCCACTTGACGGGGTC	
**11-12R**	ATAA*GGATCC*ACCCTCTCCTTTGTGACCCC	698
**15 F**	TAAA*CTCGAG*AGTACACGGTTCTTTGGGAGC	
**15 R**	ATTA*TCTAGA*TCTCCCTCCTGGTGTTTCCC	584
**21-23 F**	TTAA*CTCGAG*AACACACATACCACCCTTCCC	
**21-23 R**	TTAA*TCTAGA*CCTGGGGACAAGGACTATTGAC	2.010
**Mutation**	*Site-directed mutagenesis*	
**p.F242S F**	TTCAAATTATCTTTTCGTATTATGTATTAGATCTTTTGTTGGAACTTGGAATGTGAAT	
**p.F242S R**	ATTCACATTCCAAGTTCCAACAAAAGATCTAATACATAATACGAAAAGATAATTTGAA	
**p.W247C F**	AGATTTTTTGTTGGAACTTGTAATGTGAATGGCCAGTCTCC	
**p.W247C R**	GGAGACTGGCCATTCACATTACAAGTTCCAACAAAAAATCT	
**p.N354H F**	TGAATAATTACTCTTGCTAACAGGGACACAAAGGTGGGGTA	
**p.N354H R**	TACCCCACCTTTGTGTCCCTGTTAGCAAGAGTAATTATTCA	
**p.G357V F**	ACAGGGAAACAAAGGTGTGGTAGCTGTGAGATTTG	
**p.G357V R**	CAAATCTCACAGCTACCACACCTTTGTTTCCCTGT	
**p.G357E F**	ACAGGGAAACAAAGGTGAGGTAGCTGTGAGATTTG	
**p.G357E R**	CAAATGTGACAGCTACCTCACCTTTGTTTCCCTGT	
**p.P407P F**	CCAAATCAGACCCTCCCACAGTTGAACATCATGAA	
**p.P407P R**	TTCATGATGTTCAACTGTGGGAGGGTCTGATTTGG	
**p.P495L F**	GAAATGCCGGGTTCTAGCCTGGTGTGACC	
**p.P495L R**	GGTCACACCAGGCTAGAACCCGGCATTTC	
**p.W497G F**	CCGGGTTCCAGCCGGGTGTGACCGAAT	
**p.W497G R**	ATTCGGTCACACCCGGCTGGAACCCGG	
**p.C498Y F**	CCGGGTTCCAGCCTGGTATGACCGAATTCTTTG	
**p.C498Y R**	CAAAGAATTCGGTCATACCAGGCTGGAACCCGG	
**p.P526S F**	CCAGCGACCACAAGTCTGTTAGCGCCCCTC	
**p.P526S R**	GAGGGCGCTAACAGACTTGTGGTCGCTGG	
**p.P526L F**	CAGCGACCACAAGCTTGTTAGCGCCCTCT	
**p.P526L R**	AGAGGGCGCTAACAAGCTTGTGGTCGCTG	
**p.A797P F**	GCACTGCTCATTTTCTTGGAACCCCTGCCAGAG	
**p.A797P R**	CTCTGGCAGGGGTCCCAAGAAAATGAGCAGTGC	
**p.A861P F**	TCAATGCCAACATGATCCGTAAGAGTGCTTCATGC	
**p.A861P R**	GCATGAAGCACTCTTACGGATCATGTTGGCATTGA	
**p.A861T F**	GTCAATGCCAACATGATCAGTAAGAGTGCTTCATGCA	
**p.A861T R**	TGCATGAAGCACTCTTACTGATCATGTTGGCATTGAC	

Underlined sequences represent the tail added to the primer that includes the restriction target (in italics)

Table S2: Mutations analyzed with bioinformatics tools

Mutation	Disease	Exon	Position[1]	NNSplice		FAS-ESS	ESE Finder	Rescue ESE	SPANR	MutPred Splice[3]	
				**5′SS**	**3′SS**	**Gained ESS[2]**	**Disrupted ESE[2]**		**PSI**	**Result**	**Confident hypothesis**
c.725T>C p.(Phe242Ser)	Lowe	9	+3	0.33	**0.87**	0	0	0	Increased	SAV	---
c.728T>C p.(Phe243Ser)	Dent-2	9	+6	0.33	**0.88**	0	0	0	---	SNV	---
c.741G>T p.(Trp247Cys)	Lowe	9	+19	0.33	**0.82**	0	0	2	Decreased	SAV	ESE Loss, ESS Gain
c.783G>C p.(Trp261Cys)	Lowe	9	-42	0.33	0.85	0	0	0	Decreased	SNV	---
c.814T>C p.(Tyr272His)	Lowe	9	-11	0.33	0.85	0	0	0	---	SNV	---
c.821T>C p.(Ile274Thr)	Dent-2	9	-4	**0.45**	0.85	0	1	0	---	SNV	---
c.952C>T p.(Arg318His)	Dent-2	11	+13	0.99	**0.14**	1	1	0	---	SNV	---
c.953G>A p.(Arg318Cys)	Dent-2	11	+14	0.99	**0.18**	0	0	0	---	SNV	---
c.962G>A p.(Gly321Glu)	Dent-2	11	+23	0.99	0.11	0	1	0	---	SNV	---
c.1001G>C p.(Arg334Pro)	Lowe	11	-56	0.99	0.11	0	0	0	---	SNV	---
c.1060A>C p.(Asn354His)	Dent-2	12	+4	0.99	**0.54**	0	1	2	---	SAV	---
c.1070G>T p.(Gly357Val)	Lowe	12	+14	0.99	**0.49**	0	0	0	---	SAV	Cryptic 5' SS
c.1070G>A p.(Gly357Glu)	Lowe	12	+14	0.99	**0.54**	1	0	0	---	SAV	ESS Loss, Cryptic 5' SS
c.1115T>G p.(Val372Gly)	Lowe	12	+59	0.99	0.43	0	0	0	---	SNV	---
c.1117A>T p.(Asn373Tyr)	Lowe	12	+61	0.99	0.43	0	0	1	---	SNV	---

Table S2: *Continuation*

Mutation	Disease	Exon	Position[1]	NNSplice 5′SS	NNSplice 3′SS	FAS-ESS Gained ESS[2]	ESE Finder Disrupted ESE[2]	Rescue ESE Disrupted ESE[2]	SPANR PSI	MutPred Splice[3] Result	MutPred Splice[3] Confident hypothesis
c.1120T>C p.(Ser374Pro)	Lowe	12	+64	0.99	0.43	0	2	0	---	SNV	---
c.1121C>T p.(Ser374Phe)	Lowe	12	+65	0.99	0.43	0	1	0	---	SNV	---
c.1123C>T p.(His375Tyr)	Lowe	12	+67	0.99	0.43	0	0	0	---	SNV	---
c.1221G>A p.(Pro407Pro)	Dent-2	12	-24	0.99	0.43	0	1	0	---	SNV	---
c.1477C>T p.(Arg493Trp)	Dent-2	15	+11	0.86	**0.76**	0	0	0	---	SNV	---
c.1484C>T p.(Pro495Leu)	Lowe	15	+18	0.86	**0.85**	2	3	0	---	SAV	ESE Loss, ESS Gain
c.1489T>G p.(Trp497Gly)	Lowe	15	+23	0.86	0.80	2	0	0	---	SAV	ESE Loss, ESS Gain
c.1493G>A p.(Cys498Tyr)	Lowe	15	+27	**0.91(ND)**	0.80	0	0	0	---	SAV	Cryptic 5' SS
c.1495G>C p.(Asp499His)	Lowe	15	+29	0.86	0.80	0	0	0	---	SNV	---
c.1498C>G p.(Arg500Gly)	Lowe	15	+32	0.86	0.80	0	0	0	---	SNV	---
c.1499G>A p.(Arg500Gln)	Lowe	15	+33	0.86	0.80	0	0	0	Decreased	SNV	---
c.1507T>C p.(Trp503Arg)	Lowe	15	+41	0.86	0.80	0	0	1	---	SNV	---
c.1523T>A p.(Val508Asp)	Lowe	15	+57	0.86	0.80	0	0	0	---	SNV	---
c.1538A>G p.(Tyr513Cys)	Lowe	15	+65	0.86	0.80	0	0	0	---	SNV	---
c.1566C>G p.(Ser522Arg)	Lowe	15	-37	0.86	0.80	0	0	0	Decreased	SNV	---
c.1567G>T p.(Asp523Tyr)	Lowe	15	-36	0.86	0.80	0	0	0	Decreased	SNV	---

Table S2: *Continuation*

Mutation	Disease	Exon	Position[1]	NNSplice		FAS-ESS	ESE Finder	Rescue ESE	SPANR	MutPred Splice[3]	
				**5'SS**	**3'SS**	**Gained ESS[2]**	**Disrupted ESE[2]**		**PSI**	**Result**	**Confident hypothesis**
c.1567G>A p.(Asp523Asn)	Dent-2	15	-36	0.86	0.80	0	0	0	---	SNV	---
c.1568A>G p.(Asp523Gly)	Lowe	15	-35	0.86	0.80	0	0	0	Decreased	SNV	---
c.1571A>G p.(His524Arg)	Lowe	15	-32	0.86	0.80	0	0	0	---	SNV	---
c.1572C>G p.(His524Gln)	Lowe	15	-31	0.86	0.80	0	1	0	---	SNV	---
c.1576C>T p.(Pro526Ser)	Dent-2	15	-27	0.86	0.80	0	0	0	---	SAV	ESE Loss
c.1576C>A p.(Pro526Thr)	Lowe	15	-27	0.86	0.80	0	0	0	---	SNV	---
c.1577C>T p.(Pro526Leu)	Lowe	15	-26	0.86	0.80	1	0	0	---	SAV	ESE Loss, ESS Gain
c.1580T>A p.(Val527Asp)	Lowe	15	-23	0.86	0.80	0	0	0	---	SNV	---
c.1598T>G p.(Ile533Ser)	Lowe	15	-5	0.86	0.80	0	0	0	---	SAV	---
c.2303T>A p.(Ile768Asn)	Lowe	21	-39	0.00	0.91	0	0	1	Decreased	SNV	---
c.2389G>C p.(Ala797Pro)	Lowe	22	+48	0.84	0.05	0	0	0	---	SNV	---
c.2418G>T p.(Glu806Asp)	Lowe	22	+52	0.84	0.05	0	1	0	Decreased	SNV	---
c.2581G>A p.(Ala861Thr)	Lowe	23	-1	**0.36**	0.49	0	0	0	Decreased	SAV	Loss of natural 5' SS
c.2581G>C p.(Ala861Pro)	Lowe	23	-1	**0.75**	0.49	0	0	0	Decreased	SAV	Loss of natural 5' SS

The NNsplices scores for the wild-type 5'/3' splice sites of exons 9, 11, 12, 15, 21, 22, and 23 are 0.33/0.85, 0.99/0.11, 0.99/0.43, 0.86/0.80, 0.00/0.91, 0.84/0.05 and 0.99/0.49, respectively.

Values that appear in bold are those that change with the mutation. SAV, Splicing Affecting Variant; SNV, Splicing Neutral Variant; ESE, Exonic Splicing Enhancer; ESS, Exonic Splicing Silencer; SS, Splice Site; ND, New Donor Site; PSI, Percentage of transcripts with the exon spliced in; (---), No predicted effect.

[1] Position of the variant in exon relative to the nearest splice site.

[2] Values 0, 1, 2 and 3 indicate the number of splicing regulatory elements gained or disrupted.

[3] Score >=0.6 corresponds to SAV; additional supporting evidence is provided by a “confidence hypothesis” which is not available for all SAVs.
